# Can plasma antioxidants prevent DNA damage in oxidative stress condition induced by growth hormone deficiency? A pilot study

**DOI:** 10.1371/journal.pone.0248971

**Published:** 2021-04-01

**Authors:** Antonio Mancini, Francesco Guidi, Carmine Bruno, Flavia Angelini, Edoardo Vergani, Paola Lanza, Alvaro Mordente, Elisabetta Meucci, Andrea Silvestrini

**Affiliations:** 1 Dipartimento di Medicina e Chirurgia Traslazionale, Università Cattolica del Sacro Cuore, Rome, Italy; 2 Fondazione Policlinico Universitario A. Gemelli IRCCS, Rome, Italy; 3 Dipartimento di Scienze biotecnologiche di base, Cliniche intensivologiche e peri-operatorie, Università Cattolica del Sacro Cuore, Rome, Italy; University of Salamanca, SPAIN

## Abstract

Adult growth hormone deficiency (GHD), a condition characterized by increased oxidative stress, is related to augmented cardiovascular, metabolic and oncological risk. A case-control observational study has been performed to evaluate DNA oxidative damage analysing the production of thymidine-glycol in lymphocytes and its correlation with plasma antioxidant levels, evaluated as Total Antioxidant Capacity (TAC). GHD was diagnosed using GHRH 50μg iv+arginine 0,5 g/Kg test, with peak GH response <9 μg/L when BMI was <30 kg/m^2^ or <4 μg/L when BMI was >30 kg/m^2^. Three groups were identified: total GHD (n = 16), partial GHD (n = 11), and controls (n = 12). Thymidine-glycol, TAC and IGF-1 have been determined respectively in lymphocytes, plasma and serum samples. When considering thymidine-glycol, we found a significant difference between total vs partial GHD and controls. Unexpectedly thymidine-glycol was lower in total GHD, also accompanied with a significant increase in plasmatic TAC. Our results showed that in adult GHD condition, the production of antioxidant species, in response to increased oxidative stress, could exert a protective effect on thymidine-glycol formation, and consequently on DNA intracellular damages. This pilot study could be inserted in the complex scenario of oxidative damage of GHD, a subtle, yet poorly defined condition, worthy of further insights.

## Introduction

Adult growth hormone deficiency (GHD) is a clinical syndrome related to low GH secretion by pituitary gland caused by congenital or acquired diseases affecting the hypothalamus-pituitary region [1]. It is largely accepted that GHD, both the childhood onset and the adult age one, is a condition characterized by increased cardiovascular risk and metabolic derangement; low-grade inflammation, in turn related to oxidative stress (OS) that may represent one of the most relevant mechanism underlying the complications, the clinical course and perhaps the mortality of such condition [2,3]. Despite adult GHD is considered a rare disease, it could be underestimated due to the largely unspecific clinical presentation; moreover, this condition is generally revealed only in patients who underwent pituitary surgery or radiotherapy for cerebral tumours, and patients who developed infectious/infiltrative diseases affecting hypothalamic-pituitary region or cranial traumas and subarachnoid hemorrhages [1]. Indeed, other pathological conditions in which GH-IGF-1 axis may be impaired include partial GHD and functional GHD either, clinically recognizable in obesity and undefined osteoporosis associated to increased fracture risk [4].

Some investigation suggest that OS causes macromolecular damage (involving lipid, protein and DNA) [5]. The main consequences of DNA oxidative damage could be impact on atherogenesis, diabetes and carcinogenesis [6,7]. It is known that high levels of oxidized DNA are present in cancer cells [8] and high generation of hydrogen peroxide is demonstrated in human tumour cells [9]. Triggered by inflammation, lympho-monocytes and epithelial cells produce reactive oxygen species (ROS) and reactive nitrogen species (RNS) with consequent oxidative and nitrative DNA lesions, implicated in inflammation-mediated carcinogenesis [10]. Furthermore, in vivo clinical trials suggest a positive role of dietary antioxidants in reducing DNA damage in cancer initiation and promotion [11,12]. One of the most mutagenic lesion in DNA is the hydroxylation of guanine in the 8-position. This process can lead to inadequate base pairing and misreading of the oxidized base and adjacent residues [6]. If repairing mechanisms cannot counteract this modification, the risk for carcinogenesis increases dramatically [11]. An indirect measure of DNA oxidation is obtained from urinary oxidized nucleosides and bases, which are water-soluble and excreted in the urine without further metabolism. An example is 8-OH-deoxyguanosine (8-OHdG), which is considered a biomarker of DNA oxidation or, more precisely, a biomarker between oxidative damage and rate of repair. It must be underlined that this parameter indicates the average rate of damage in whole body and therefore is different from the determination of oxidized bases in specific tissue or cell types. Moreover, the quantification of 8-OHdG can be determined by ELISA method [13] that require tedious assay procedure, and sometimes inadequate level of sensitivity. Recently, an alternative marker of DNA oxidation is reported, the Thymidine-glycol (ThyG) (5,6-dihydro-5,6-dihydroxy-2’-deoxythymidine). This molecule is produced when thymidine is modified by hydroxyl radicals [14]. ThyG is considered a more specific marker since it is not incorporated in RNA, furthermore while 8-OH-dG is rapidly excised from DNA and excreted in the urine [15], ThyG remains in the tissues, thus representing a more appropriate marker for oxidative DNA damage *in vivo* [16]. As tissue DNA damage marker, it has been previously evaluated in patients affected by primary lung cancer [16].

Since the early identification of oxidative DNA damage may represent a possible strategy to counteract the appearance of the above-mentioned comorbidity, firstly we aimed to evaluate the entity of DNA oxidative damage applying the immune-histochemical method for evaluation of ThyG in lymphocytes of adult GHD patients. Secondly, we aimed to correlate plasma antioxidant levels, evaluated as total antioxidant capacity, as previously reported [2], and ThyG evaluations.

## Materials and methods

The patients involved in this study consisted of 39 subjects (21 males, 18 females), admitted to the University Hospital “Policlinico Gemelli” Dept. of Translational Medicine and Surgery, and enrolled (from January 2016 to October 2018) after being given explanation of purposes and nature of the study, conducted in accordance with the declaration of Helsinki, as revised in 2013. The study protocol was approved by “Fondazione Policlinico Gemelli” ethical committee. After being given a written consent, previously waived by the ethical committee, GHD untreated patients and controls of both sexes were included in the study. Demographic parameters are reported in Table 1. They were part of the same cohort of patients enrolled for a previous study [2].

**Table 1:** Demographic and anthropometric parameters.

	Total GHD	Partial GHD	Controls
**Age (years)**	50.94±2.91	50.54±4.12	46.36±4.58
**BMI (kg/m**^**2**^**)**	27.04±1.92	26.22±2.13	24.19±2.10
**Ethnicity**	All caucasian	All caucasian	All caucasian
**M**	9	5	7
**F**	7	6	5

Exclusion criteria were: age under 18 or over 70; obesity of genetic origin or related to other endocrine diseases; history of cranial hypertension or active cranial hypertension, decompensated type 1 or 2 diabetes mellitus; autoimmune diseases under immunosuppressive treatment; corticosteroid treatment (except for topic, inhalatory and oral hydrocortisone as replacement regimen); other diseases characterized with low insulin-like growth factor-1 (IGF-1) such as liver disease, malabsorption and malnutrition; active malignancy, acute organ failure (such as renal, hepatic or heart acute failure), smoking, cardiovascular disease or acute diseases. No patients received GH therapy at the study enrolment.

GHD was diagnosed with dynamic test, using Growth Hormone-Releasing Hormone (GHRH) 50 μg intravenous + arginine 0,5 g/Kg (GHRH/ARG). Patients were divided in three groups, according to AACE guidelines cut-off [17].

Upon 16 patients, 9 males and 7 females, presented a peak GH response < 9 ng/ml (or < 4 ng/ml when BMI ≥ 30 kg/m^2^) and were classified as total GHD group (t-GHD); 11 patients, 5 males and 6 females, with GH peak between 9 and 16 ng/ml were considered as partial GHD group (p-GHD). Finally, 12 subjects, 7 males and 5 females, matched for age and sex, with GH peak > 16 ng/ml were included as control group (CTRL).

Within the total GHD group, different aetiologies were identified: 6 cases of primary empty sella, 6 cases of idiopathic isolated GHD, 1 case of Arnold-Chiari syndrome, 1 case of Sheehan’s syndrome, 1 case of post-surgical hypopituitarism, 1 case of pituitary infarction.

Age in t-GHD group ranged from 37 to 70 years, in p-GHD group from 24 to 70 years and from 23 to 70 years in CTRL group. Median ± interquartile BMI were 27.14 ± 2.85 kg/m^2^, 26.23 ± 8.36 kg/m^2^ and 22.51 ± 1.26 kg/m^2^ in t-GHD, p-GHD and control respectively.

Patients underwent venous sampling at 8 a.m., after an overnight fasting, collecting blood samples by three pyrogen-free tubes, the first for TAC with heparin as anticoagulant, the second for ThyG with EDTA as anticoagulant, the third for serum evaluation without anticoagulant.

TAC was evaluated by spectrophotometric method, with a modification of the method developed by Rice-Evans and Miller [18], as previously described [19]. The method is based on the antioxidants inhibition of the absorbance of the radical action 2,2I-azinobis (3-ethylbenzothiazoline-6 sulphonate) (ABTS^.+^) formed by interaction between ABTS (150 μM) and ferrylmyoglobin radical species, generated by activation of metamyoglobin (2.5 μM) with H_2_O_2_ (75 μM). Aliquots of the frozen plasma were thawed at room temperature and 10 μl of the samples were tested immediately. The manual procedure was used with only minor modifications, i.e. temperature at 37°C to be in more physiological conditions and each sample assayed alone to carefully control timing and temperature. The reaction was started directly in cuvette through H_2_O_2_ addition after 1 min equilibration of all other reagents (temperature control by a thermocouple probe, model 1408 K thermocouple, Digitron Instrumentation Ltd, Scunthorpe, United Kingdom) and followed for 10 min under continuous stirring, monitoring at 734 nm, typical of the spectroscopically detectable ABTS^.+^. The presence of chain-breaking antioxidants induces a lag time (the Lag phase) in the accumulation of ABTS^.+^ whose duration is proportional to the concentration of this type of antioxidants. Antioxidant capacity afforded by chain-breaking antioxidants is expressed as length of Lag phase (LAG, sec). Trolox, a water-soluble tocopherol analogue, was used as a reference standard and assayed in all experiments to control the system. Absorbance was measured with an Agilent 8453 UV/Vis spectrophotometer (Palo Alto, CA, USA) equipped with a cuvette stirring apparatus and a constant temperature cell holder. Measurements of pH were made with a PHM84 Research pH meter (Radiometer, Copenhagen, Denmark); the electrode response was corrected for temperature. Unless otherwise stated, experiments were repeated two to three times; qualitatively similar results were obtained with individual values varying <8%. In the Lag mode, the assay mainly measures non-proteic and non-enzymatic antioxidants that are primarily extracellular chainbreaking antioxidants, such as ascorbate, urate and glutathione.

Lympho-monocyte population was collected by centrifugation (1200 rpm for 30 minutes) on a density gradient, then frozen in FBS with 10% of DMSO in liquid nitrogen. After gathering all the samples, the collected cells were quickly thawed and washed twice in 10% FBS medium. They were subsequently suspended in PBS (w/o Ca^2+^ and Mg^2+^) and then counted. Given that, about 1000 cells/200 μl of PBS were taken and spotted onto a positively charged slide (Dako) by cytocentrifuge (Cytospin3-Shandon Thermo Fisher Scientific) at 500 rpm for 5 min. After drying the slides on air for 30 minutes, the samples were fixed in 4% PFA for 7 minutes. Subsequently, the cells, after a washing in PBS, were treated with 0.5% TritonX (5 minutes) to permeabilize the membranes and afterwards with 3% H_2_O_2_ for 5 minutes, in order to inhibit endogenous peroxidases. Monoclonal Anti-Thymidin-Glycol antibody was purchased by jaICa, Japan (catalog No. MNW-020P, lot No. 001 MNW-020P). The antibody was incubated for 30 minutes at RT in a wet chamber at dilution 1:40 in diluent (Dako). After consecutive washes the EnVision^TM^ Flex/HRP (Dako) polymer was used as secondary antibody for 20 minutes at RT. In each staining cycle, a negative control was included, omitting the primary Ab, to evaluate any non-specific staining. The chromogen DAB was used to evidence positivity through the formation of brown precipitates. The results were examined and interpreted with an optical microscope by three readers independently, who gave, respectively, a score from 1 to 5; the mean of the three evaluations was considered for each sample.

Serum concentrations of IGF-1 were evaluated using immunechemiluminometric assays on a Roche Modular E170 analyser (Roche Diagnostics, Indianapolis, IN, USA). The intra- and inter-assay CV were, respectively, < 5.0% and < 7.0%.

### Statistical analysis

Statistical analysis was performed using Stata 13 software. The planned sample size of minimum 10 cases for each group was not based on formal power nor effects estimates, due to the lack of any information on the size of the minimal difference worth detecting for each of the study parameters. Instead, the sample size was established based on considerations of statistical practicality due to the rarity of GHD condition. With this sample size, the study has power >80% to detect any differences between cases and controls, that is as large or greater than 75% of the standard deviation of that specific parameters. Considering that the comparison concerns differences between diseased subjects and healthy controls in physiologic parameters of oxidative stress, smaller differences are of little, if any, interest. Median and interquartile range were used to describe quantitative variables. According to D’Agostino and Pearson test, variables did not follow standard distribution. The statistical analysis was carried out using the Mann-Whitney U-Test to study the differences among groups. The level of significance has been set at 0.05.

## Results

**Fig 1:**
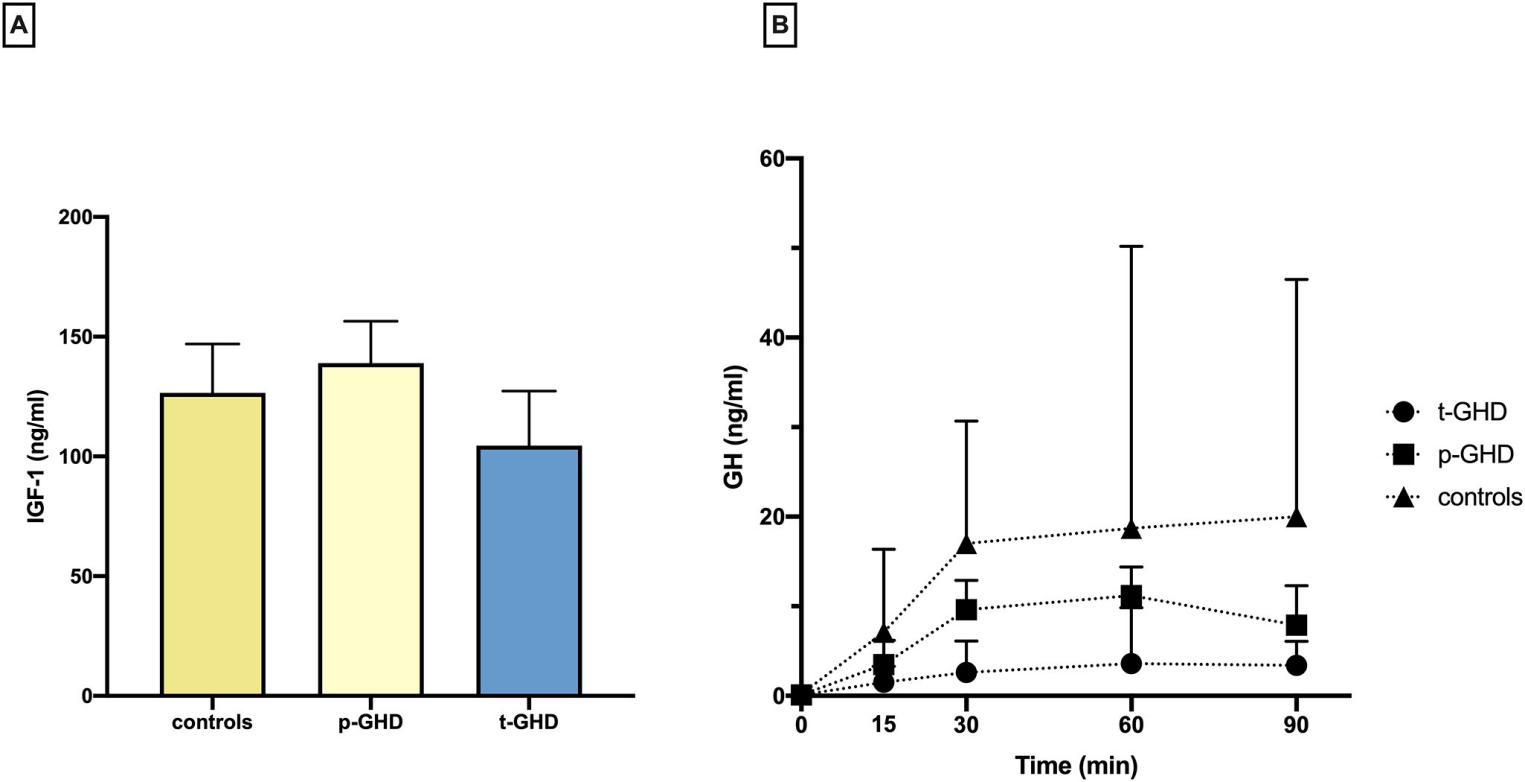
IGF-1 values and GHRH + arginine test. Panel A: Median ± interquartile range of IGF-1 values (ng/ml) in the three groups; panel B: Median ± interquartile range of GH values (ng/ml) at baseline and after 15, 30, 60, 90 minutes after iv administration of GHRH + arginine in the three groups.

Fig 1 shows the median ± interquartile values of GH response to GHRH/ARG test in the three groups: CTRL, p-GHD and t-GHD (left panel); in the right panel it is depicted IGF-1 median ± interquartile levels. As expected, IGF-1 levels and GH peak were significantly lower in t-GHD group.

**Fig 2:**
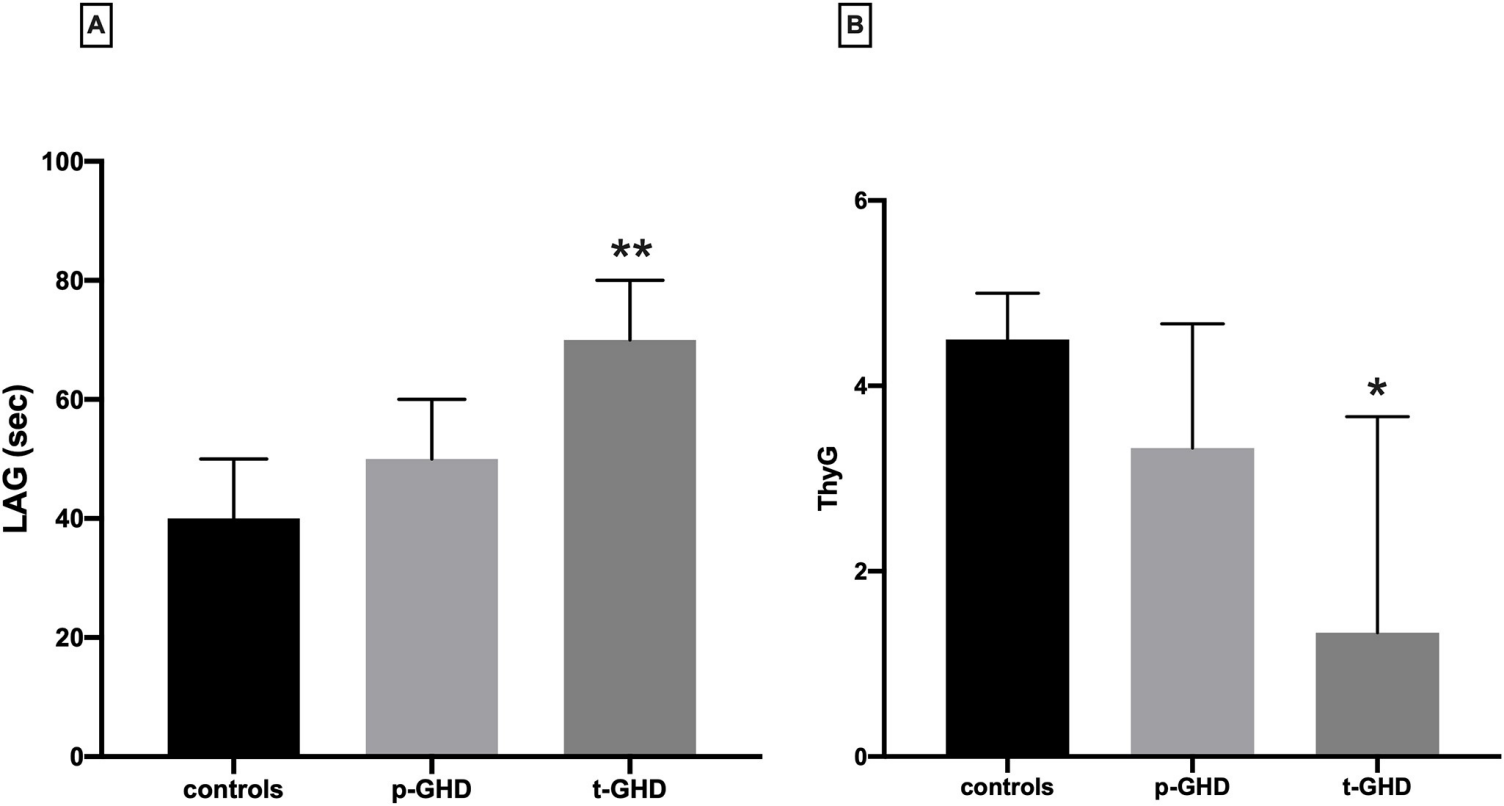
LAG and ThyG levels in the three groups. Panel A: median ± interquartile range of TAC, expressed by LAG (sec), in the three groups. **p<0.05 vs p-GHD and controls; panel B median ± interquartile range of ThyG in the three groups (*p<0.05 vs controls).

TAC has been expressed in LAG phase, as specified in materials and methods section. The median ± interquartile ranges of LAG (panel A) and ThyG (panel B) in the three groups are depicted in Fig 2. The LAG values in t-GHD significantly differed from p-GHD (p = 0.002) and CTRL (p = 0.0001). When considering ThyG, we found a significant difference between t-GHD vs control groups (p = 0.04). Partial GHD showed a clear trend toward intermediate levels, even though not significant when compared with the other two groups.

**Fig 3:**
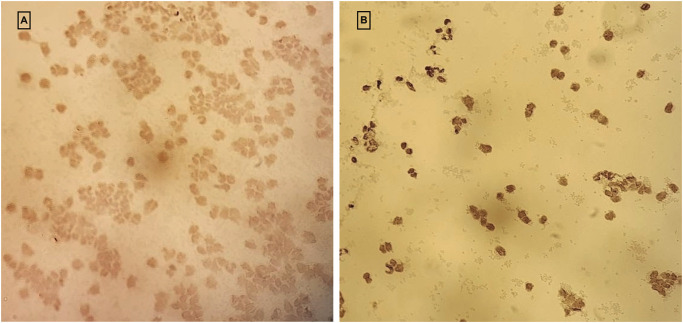
Evaluation of Thymidine-glycol in lymphocytes. A) example of a patient with a low-grade score (1) and B) example of patient with a high-grade score (5) of anti-Thymidine-glycol. Details of the method are reported in material and methods section. The images here represented have been taken on 20x magnification.

Fig 3 exemplifies the differential expression of ThyG in two patients, representing or very low (left panel) or intense (right panel) positivity.

## Discussion

This is the first evaluation of ThyG in lympho-monocytes, a sample that appears easier to obtain than other human tissues, which require biopsy sampling. Moreover, this is the first evaluation of ThyG as marker of DNA damage in adult GHD, a rare condition affected by oxidative stress. Despite this method employs a brief exposure to H_2_O_2_ which could influence the results, all samples were treated identically. Unexpectedly, products of DNA oxidation were lower in GHD, even though this datum was combined with an increase in plasmatic antioxidant capacity.

In a previous study we did not find significantly different levels of LAG between GHD and control subjects, however a different antioxidant pattern was recognized, also when compared with other condition of insulin-resistance [20]. Extending our studies, we found significantly higher LAG in GHD vs controls, also associated with significantly higher levels of indexes of low-grade inflammations, such as free-light chains of immunoglobulins [21]. The evaluation of macromolecular oxidation allowed us to suppose an increase of antioxidants in total, but also in partial GHD conditions. However, surprisingly, a marker of DNA damage such as urinary 8OH-dG was not different between GHD conditions and controls [2].

Furthermore, conflicting evidences concerning GH effects on antioxidant levels are reported. In some models, GH seems to exert a pro-oxidant activity; it increases ROS production in monocyte cells [22] and in neutrophils, in the last its effect is coupled with inhibition of apoptosis mediated by down-regulation of Fas expression [23]. On the contrary, in other models an antioxidant effect is prevalent as evidenced by two-week treatment with GH and insulin in Wister rats; indeed this treatment decreases ROS generation and oxidative DNA damage in heart mitochondria, but at the same time in the liver decreased mitochondrial ROS generation but increase oxidative damage in mt-DNA [24]. Knock-out mice for GH receptor, revealed an increase in antioxidant defences related to sex [25]. After 1 year of GH replacement therapy in prepubertal children affected by GHD condition is reported an increase in antioxidant defences with a normalization of oxidative stress [26]. In adult GHD condition a short-term GH administration with a dose able to increase IGF-I levels can revert the augmented lipid peroxidation [27]. Furthermore, was found that low dose GH therapy can ameliorate relation between OS and insulin resistance in GHD condition also improving insulin sensitivity [28,29]. We have recently reported that although if adult GHD condition shares with metabolic syndrome some biochemical features, in these illnesses can be evidenced two different pattern of plasma antioxidant capacity and coenzyme Q10 levels [20,21].

In effort to clarify, other indexes of macromolecular damage have been investigated such as nitro-tyrosine and carbonyls groups as markers of protein oxidation but these parameters seemed not to be affected by GHD condition [30]. Furthermore, a significant increase in oxidized LDL, in turn associated to a worse atherosclerotic picture compared to healthy controls, may be detected in such condition [27,30,31]. On the contrary, in a randomized double-blind placebo-controlled study, GH replacement therapy in adult GHD is reported to reduce lipid peroxidation, assessed in plasma by lipid hydroperoxides, and LDL susceptibility to peroxidation [32]. Other authors reported that oxidized LDL levels should be interpreted considering the duration of GHD disease proposing a solution to this apparent paradigm [30]. More recently, a study reported an increase of oxidative stress and endothelial dysfunction that could be present in early stages of GHD condition also influenced by time-course of the disease. Moreover, this study described an increase in oxidized lipoproteins production associated to a lower values of reduced/oxidized glutathione ratio (GSH/GSSG) and a worse endothelial function in adult GHD corroborating that previously reported [31,33].

To the best of our knowledge, no data are reported on DNA damage, evaluated by ThyG analysis, in GHD condition, which could have some oncological outcomes. Despite in vitro and in vivo studies apprise evidence to relation between GH-IGF-1 and cancer risk and the concern for a possible role in cancer progression by GH administration, a recent meta-analysis suggests that GH replacement therapy reduces cancer risk in adults GHD [34]. Moreover, overall epidemiological evidences show lower tumour prevalence in treated versus untreated GHD patients [35]. Thus, the rationale for an augmented oncological risk in GHD condition could be oxidative DNA damage.

According to our previous studies, LAG values showed a progressive increase from controls to partial GHD to total GHD, suggesting a progressively greater oxidative stress and concomitantly compensatory rise in antioxidant systems [20]. As previously reported, LAG is a time-dependent index of non-proteic non-enzymatic chain breaking molecules, such as urate, ascorbate, vitamin E, therefore mainly extracellular [19,36,37]. However, chain breakers are presumably related to intracellular antioxidants, since gene regulation of antioxidants is elicited by oxidative stress; in some case, both intra- and extracellular activity is exerted by some molecules such as Coenzyme Q10, which has been already evaluated in our previous study in GHD patients [20]. As a next step in this process, we aimed to examine ThyG, as a parameter of oxidative DNA damage, and then correlate it with GH production to evaluate if plasma antioxidants can prevent DNA damage in OS condition induced by GHD condition. The above exposed opposite pattern of LAG and ThyG may corroborate in favour of this hypothesis.

Nevertheless, the data reported here as a case-control observational study possess some limitations, therefore it deserves to be confirmed in a larger population study. A limit of this pilot study is that we have not evaluated intracellular enzymes involved in response to oxidative stress. Moreover, we focus this pilot study to few parameters and ascertained only an association between TAC and ThyG with inverse pattern; however, we cannot establish a causal relation.

Finally, if confirmed in a larger population, our data suggest that the ThyG could be a useful biomarker of OS in GHD condition, differently from 8-OH-dG, which did not differ in GHD vs control groups [2], and that despite increase in oxidation of other macromolecular classes, DNA seems to be preserved by circulating antioxidants.
